# Rock outcrop orchids reveal the genetic connectivity and diversity of inselbergs of northeastern Brazil

**DOI:** 10.1186/1471-2148-14-49

**Published:** 2014-03-15

**Authors:** Fábio Pinheiro, Salvatore Cozzolino, David Draper, Fábio de Barros, Leonardo P Félix, Michael F Fay, Clarisse Palma-Silva

**Affiliations:** 1 Instituto de Botânica, Núcleo de Pesquisa do Orquidário do Estado, Avenida Miguel Estéfano 3687, 04301-012 São Paulo, SP, Brazil; 2 Dipartimento di Biologia, Complesso Universitario di Monte S. Ângelo, Università degli Studi di Napoli Federico II, 80100 Napoli, Italy; 3 Institute for Plant Protection, Consiglio Nazionale delle Ricerche, Via Madonna del Piano 10, I-50019 Sesto Fiorentino (FI), Italy; 4 Departamento de Ciencias Naturales, Universidad Técnica Particular de Loja, San Cayetano Alto s/n, CP 11 01 608 Loja, Ecuador; 5 Departamento de Fitotecnia, Laboratório de Citogenética Vegetal, Universidade Federal da Paraíba, Areia, PB, Brazil; 6 Jodrell Laboratory, Royal Botanic Gardens, Kew, Richmond, Surrey TW9 3DS, UK; 7 Laboratório de Ecologia Molecular, Departamento de Ecologia, Universidade Estadual Paulista, 13506-900 Rio Claro, SP, Brazil

**Keywords:** Brazilian Atlantic Forest, Caatinga, Chloroplast microsatellites, *Epidendrum cinnabarinum*, *Epidendrum secundum*, Genetic drift, Nuclear microsatellites, Orchidaceae

## Abstract

**Background:**

Because of their fragmented nature, inselberg species are interesting biological models for studying the genetic consequences of disjoint populations. Inselbergs are commonly compared with oceanic islands, as most of them display a marked ecological isolation from the surrounding area. The isolation of these rock outcrops is reflected in the high number of recorded endemic species and the strong floristic differences between individual inselbergs and adjacent habitats. We examined the genetic connectivity of orchids *Epidendrum cinnabarinum* and *E. secundum* adapted to Neotropical inselbergs of northeastern Brazil. Our goals were to identify major genetic divergences or disjunctions across the range of the species and to investigate potential demographic and evolutionary mechanisms leading to lineage divergence in Neotropical mountain ecosystems.

**Results:**

Based on plastid markers, high genetic differentiation was found for *E. cinnabarinum* (*F*_ST_ = 0.644) and *E. secundum* (*F*_ST_ = 0.636). Haplotypes were not geographically structured in either taxon, suggesting that restricted gene flow and genetic drift may be significant factors influencing the diversification of these inselberg populations. Moreover, strong differentiation was found between populations over short spatial scales, indicating substantial periods of isolation among populations. For *E. secundum*, nuclear markers indicated higher gene flow by pollen than by seeds.

**Conclusions:**

The comparative approach adopted in this study contributed to the elucidation of patterns in both species. Our results confirm the ancient and highly isolated nature of inselberg populations. Both species showed similar patterns of genetic diversity and structure, highlighting the importance of seed-restricted gene flow and genetic drift as drivers of plant diversification in terrestrial islands such as inselbergs.

## Background

Inselbergs are isolated rock outcrops typically harboring rupicolous vegetation and embedded within a landscape composed of contrasting plant communities. Because of their disconnected geographic nature, inselbergs are frequently compared with oceanic islands, since most of them display a marked ecological isolation from the surrounding area [1]. The isolation and ancient age of these rock outcrops is reflected in the high number of recorded endemic species (reviewed by [2]) and the strong floristic differences observed between individual inselbergs and their surrounding habitats [3,4]. Some authors have also suggested that inselbergs may have acted as refugia for xerophilic or cold-adapted species during glacial/interglacial cycles [4,5]. In addition, most inselbergs maintain their typical attributes irrespective of geographic location, enabling broad-scale comparisons even between different and contrasting biomes, such as mesic forests and seasonally dry plant communities [2]. As biological islands, inselbergs are promising ecosystems for biogeographic and evolutionary studies, comparable to their oceanic counterparts [6].

Phylogeographic studies of inselberg-adapted species have provided insights into evolutionary processes leading to diversification of lineages and species [7-11]. Such studies have uncovered strong phylogeographic structure, high population differentiation and extensive genetic diversity levels, supporting the view of inselbergs as centers of species diversity and endemism. Species occurring in small isolated patches are expected to experience reduced gene flow, significant genetic drift and high levels of population divergence [6]. Indeed, genetic studies of inselberg species have provided strong support for these expectations [7,12-15]. In isolated populations, gene flow is constrained, and genetic drift is expected to be the predominant force governing allele frequencies [8,16].

Porembsky et al. [17] have noted that plant community organization on rock outcrops is driven by stochastic colonization events. Thus, phylogeographic studies of multiple co-occurring species with similar biological traits (e.g. pollination and seed dispersion) may be particularly useful for understanding the role of stochasticity (i.e. genetic drift) in the evolution of these naturally fragmented populations. By comparing the phylogeographic structures of different species, one can infer whether the current plant community has been historically stable as evidenced by geographically similar genetic structure. Alternatively, if species distributions are ephemeral over evolutionary time and more influenced by intrinsic species preferences, a mixture of phylogeographic structures is expected [18,19].

Phylogeographic studies focused on organisms associated with mesic forest communities have indicated a strong association between the occurrence of glacial cycles and the fragmentation of forest-dwelling species, supporting the classical model of tropical refugia [20-22]. On the other hand, species associated with open biomes, such as grassland, savannas and dry forests, show variable demographic responses to past glacial cycles, suggesting a more complex scenario [21,23]. In this regard, cross validation of environmental envelope models (EEMs according to terminology suggested by [24]) and molecular genetic data have confirmed different past demographic scenarios for a wide array of species. Fragmentation [25], expansion [26] and long-term persistence [27] have been detected in organisms associated with open vegetation communities. By combining EEMs and molecular genetic tools, different phylogeographic hypotheses depicting the role of past climatic fluctuations in lineage diversification can be tested.

The Caatinga is the largest, most diverse dry seasonal tropical forest biome in the Neotropical region [28]. Despite the extensive geographic area of this biome, relatively few phylogeographic studies have been conducted using species from the Caatinga [27,29-32]. This paucity of studies precludes broad conclusions regarding the impact of past climate oscillation events on the genetic structure of organisms. The high number of endemic species [28] coupled with old lineage-divergence times [27,33,34] implies an ancient origin for Caatinga communities. In addition, paleoclimate models [35] indicate that dry forest distribution within the Caatinga was stable during the Last Glacial Maximum (LGM). Indeed, high species diversity and floristic differences have been found among Caatinga rock outcrops [3,36], supporting the hypothesis of inselbergs as ancient, stable refuges of diversity [4,5].

In the present study, we used nuclear and plastid microsatellite markers to analyze phylogeographic structure and genetic diversity of *Epidendrum cinnabarinum* and *E. secundum* (Orchidaceae), two widespread, co-occurring species commonly found on inselbergs in northeastern Brazil. *Epidendrum cinnabarinum*, a polyploid species with 2*n* = 240 chromosomes [37], occurs in Caatinga and Brazilian Atlantic Forest (BAF) inselbergs and on sand dune vegetation along the seashore in northeastern Brazil. *Epidendrum secundum* exhibits a diploid chromosome count of 2*n* = 56 in most populations [37]; it has a much broader geographic distribution, occurring in Central America, Guiana and Andean ranges and the Brazilian Central Shield [38]. This species occurs preferentially on rock outcrops, with abundant populations observed on Caatinga and BAF inselbergs. In this study, we also applied EEMs to explore the demography of both species during important climatic oscillation events: the LGM (21,000 ka) and Last Interglacial (LIG; 120,000 ka). The following specific questions were addressed: (1) What is the current extent of genetic structure and the degree of isolation among disjoint populations on inselbergs? (2) Were the current range distributions of both species stable during Late Quaternary climatic oscillations? and (3) Are there phylogeographic breaks separating populations from different biomes (Caatinga and BAF) and ecoregions (Chapada Diamantina and Planalto da Borborema)? We also considered the phylogeographical and genetic structure of *E. cinnabarinum* and *E. secundum* in light of results based on climate modeling, paleovegetation reconstructions and the island-like nature of populations distributed on inselbergs.

## Results

### Plastid and nuclear genetic diversity of sampled populations

**Figure 1:**
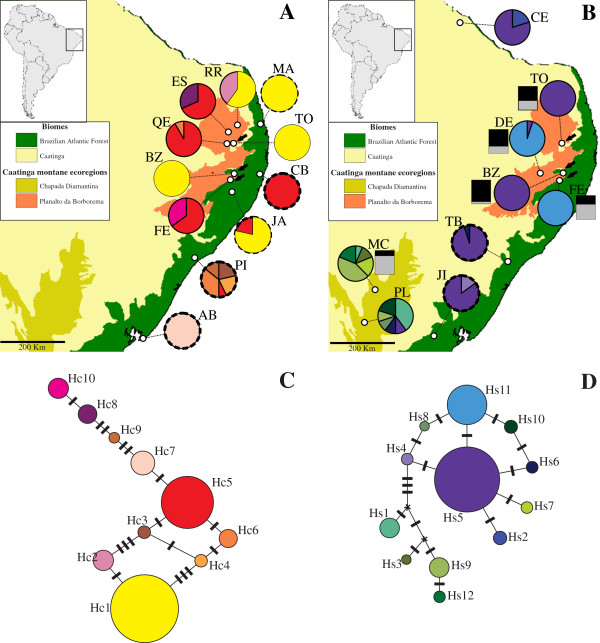
**Geographic distribution of sampled populations.** Maps showing sampled populations of *Epidendrum cinnabarinum***(A)** and *E. secundum***(B)**, and respective plastid DNA networks for each species **(C and D)**. Pie charts reflect the frequency of occurrence of each haplotype in each population. Haplotype colours correspond to those shown in networks. Pie charts with solid and broken outlines indicate Caatinga and Brazilian Atlantic Forest populations, respectively. Nuclear genetic groups are indicated for *E. secundum* (Nuclear cluster 1 – black; Nuclear cluster 2 – grey). In the statistical parsimony networks **(C and D)**, the haplotype frequencies are proportional to circle sizes. The number of mutations required to explain transitions among haplotypes is indicated along the lines connecting the haplotypes by cross hatches.

Analysis of seven plastid loci recovered a total of 10 haplotypes for *E. cinnabarinum* and 12 haplotypes for *E. secundum* (Figure 1C, 1D, Table 1, Additional file 1: Table S1), with no haplotype sharing between species. One to five haplotypes were found within *E. cinnabarinum* populations, while one to six haplotypes were detected within *E. secundum* populations. Similar diversity levels were detected for both species concerning haplotype richness (*E. cinnabarinum* 0.000–3.066; *E. secundum* 0.000–4.873) and haplotype diversity (*E. cinnabarinum* 0.000–0.813; *E. secundum* 0.000–0.800) (Table 1). In two sympatric populations, TO and BZ, genetic diversity parameters were identical for both species (Table 1).

**Table 1:** **Genetic characterization of populations of *Epidendrum cinnabarinum *** **and *E. secundum *** **based on plastid markers**

		**Plastid loci**		
**Population**	**ID**	**NH**	**HR**	**HD**
*Epidendrum cinnabarinum*				
Mamanguape	MA	1	0.000	0.000
Serraria	RR	2	0.990	0.505
Esperança	ES	2	0.966	0.456
Queimadas	QE	2	0.583	0.167
Jaqueira	JA	2	0.904	0.363
Cabo de Santo Agostinho	CB	1	0.000	0.000
Pirambu	PI	5	3.066	0.813
Abaeté	AB	1	0.000	0.000
*Epidendrum secundum*				
Maranguape	CE	2	1.000	0.337
Brejo da Madre de Deus	DE	2	0.800	0.100
Serra de Itabaiana	TB	2	0.800	0.100
Morro do Chapéu	MC	5	4.000	0.767
Palmeiras	PL	6	4.873	0.800
Serra da Jibóia	JI	2	0.996	0.268
Sympatric				
Pedra de Santo Antonio (*E. cinn.*)	TO	1	0.000	0.000
Pedra de Santo Antonio (*E. sec*.)		1	0.000	0.000
Bezerros (*E. cinn.*)	BZ	1	0.000	0.000
Bezerros (*E. sec*.)		1	0.000	0.000
Camocim de São Félix (*E. cinn.*)	FE	2	0.978	0.479
Camocim de São Félix (*E. sec*.)		1	0.000	0.000

Identification code of sampled populations (ID), number of haplotypes (NH), haplotype richness (HR) and haplotype diversity (HD) for seven loci.

With respect to six nuclear markers genotyped in *E. secundum*, moderate levels of genetic diversity were observed in most populations (Table 2). The number of alleles per population ranged from 19 to 23, and allelic richness per population varied from 2.28 to 2.77. Expected heterozygosity per population ranged from 0.442 to 0.608. One to nine private alleles were observed within populations, with values of private allelic richness ranging from 0.11 to 0.72. Inbreeding coefficients, which ranged from -0.032 to 0.156, were not significantly different from zero in any population. The presence of genotyping errors due to stuttering or null alleles (frequencies ranging from 0 to 0.09, estimated using Brookfield Equation one) was ruled out using MICRO-CHECKER tests [39].

**Table 2:** **Genetic characterization of populations of *Epidendrum secundum *** **based on nuclear markers**

**Populations/Code**	**A**	**PA**	**AR**	**PAR**	***H***_**E**_	***f ***^**1**^	***M-*** **ratio**^**2,3**^
Pedra de Santo Antonio/TO	19	1	2.28	0.11	0.462	0.156	0.872
Brejo da Madre de Deus/DE	22	0	2.35	0.19	0.466	0.073	0.895
Bezerros/BZ	20	2	2.29	0.22	0.442	-0.065	0.785
Camocim de São Félix/FE	23	9	2.39	0.35	0.459	0.154	0.794
Morro do Chapéu/MC	22	11	2.77	0.72	0.608	-0.032	0.875

^1^Departures of within-population inbreeding coefficients (*f*) from HWE were not detected; ^2^A population is considered to have undergone a bottleneck if its *M*-ratio value falls below the lower threshold of critical *M*-ratio (*M*c = 0.625) showed on Table 4; ^3^No bottlenecks detected.

Populations sampled, number of alleles (A), number of private alleles (PA), allelic richness (AR), private allelic richness (PAR), expected heterozygosity (*H*_E_), the within population inbreeding coefficient *f* and the *M*-ratio values for six nuclear loci.

### Genetic structure of *E. cinnabarinum*

A haplotype network based on plastid markers showed loops (ambiguities), and no apparent geographic structure was observed (Figure 1C). The most frequent haplotype in both BAF and Caatinga biomes (Hc1) was found in 46.8% of individuals and in 5 out of 11 sampled populations. Hc5, the second most frequent haplotype, was also distributed in both biomes and occupied a central position in the haplotype network (Figure 1C). Only 3 (Hc1, Hc5 and Hc6) out of 10 haplotypes were found to occur in more than one population.

High genetic differentiation was observed across populations, with *F*_ST_ = 0.644 and *G*_ST_ = 0.672. According to an analysis of molecular variance (AMOVA) (Table 3), a high proportion of the genetic variability in the haplotype data resided among populations (64.41%, P < 0.0001), with only 35.59% accounted for within populations. Hierarchical AMOVA did not support a division between Caatinga and BAF populations (Table 3; *P* = 0.704). Pairwise *F*_ST_ comparisons among populations (Additional file 2: Table S2) ranged from 1.000 to 0.000, with most values found to be significant (*P* < 0.005). Haplotype differentiation between populations separated over short spatial scales was observed between populations QE and TO (12 km apart) and between BZ and FE (18 km apart) (Additional file 2: Table S2, Figure 1A). Other than the strong genetic differentiation found among populations, no among-population phylogeographic structure or isolation by distance was detected (*P* = 0.114 and *P* = 0.084, respectively).

**Table 3:** **Analysis of molecular variance (AMOVA) for plastid microsatellite data for *Epidendrum cinnabarinum *** **populations, using two different models**

**Source of variation**	**d.f.**	**Variance components**	**Variation (%)**	***P*-value**
1) Among populations	10	0.23999	64.41	*P* < 0.001
Within populations	166	0.13262	35.59	
2) Between Biomes (Caatinga and Brazilian Atlantic Forest)	1	-0.03251	-9.10	*P* = 0.704
Among populations within biomes	9	0.25725	71.99	*P* < 0.001
Within populations	166	0.13262	37.11	*P* < 0.001

One model includes all populations pooled, and the other includes populations from different biomes (Caatinga and Brazilian Atlantic Forest) separated.

### Genetic structure of *E. secundum*

Analysis of plastid markers yielded a network lacking clear geographic structuring (Figure 1D). Haplotype Hs5 was shared between populations from Caatinga and BAF biomes, and also between Chapada Diamantina and Planalto da Borborema ecoregions (Figure 1B, 1D). Hs5 was also the most frequent haplotype, found in 54.5% of individuals and in six out of nine sampled populations. Seven haplotypes were restricted to Chapada Diamantina populations (Figure 1B).

The only hypothesis of population differentiation significantly supported by AMOVA was that between populations from Chapada Diamantina and the remaining localities (Table 4). The partitioning of plastid genetic diversity and structure among *E. secundum* populations was also very similar to the patterns observed for *E. cinnabarinum*. AMOVA results indicated that a high proportion of uncovered genetic variability was found among populations (63.64%, *P* < 0.0001), with only 36.36% attributed to within-population variability (Table 4). Genetic differentiation was high across all populations, with *F*_ST_ = 0.636 and *G*_ST_ = 0.632. As observed for *E. cinnabarinum*, contrasting haplotypes were detected between populations BZ and FE. Pairwise *F*_ST_ comparisons among populations ranged from 1.000 to 0.000, with most values significant (*P* < 0.005; Additional file 3: Table S3). No phylogeographic structure was detected among populations, as *R*_ST_ was not significantly larger than *F*_ST_ (*P* = 0.503). Isolation by distance among populations was also not detected (*P* = 0.822).

**Table 4:** **Analysis of molecular variance (AMOVA) for nuclear and plastid microsatellite data for *Epidendrum secundum *** **populations, using four different models**

**Source of variation**	**d.f.**	**Variance components**	**Variation (%)**	***P*-value**
*Nuclear microsatellite*				
1) Among populations	4	0.97604	4.58	*P* = 0.087
Within populations	187	20.33329	95.42	
2) Between Chapada Diamantina and Planalto da Borborema ecoregions	1	1.75359	7.81	*P* = 0.205
Among populations within biomes	3	0.36609	1.63	*P* = 0.331
Within populations	187	20.33329	90.56	*P* = 0.098
*Plastid microsatellite*				
1) Among populations	8	0.22274	63.64	*P* < 0.001
Within populations	165	0.12727	36.36	
2) Between Biomes (Caatinga and Brazilian Atlantic Forest)	1	0.00302	0.86	*P* = 0.386
Among populations within biomes	7	0.22154	62.97	*P* < 0.001
Within populations	165	0.12727	36.17	*P* < 0.001
3) Between Chapada Diamantina and Planalto da Borborema ecoregions	1	0.05130	11.61	*P* = 0.201
Among populations within biomes	4	0.25806	58.42	*P* < 0.001
Within populations	108	0.13241	29.97	*P* < 0.001
4) Between Chapada Diamantina and remaining populations	1	0.85932	61.21	*P* < 0.05
Among populations within groups	7	0.22273	15.87	*P* < 0.001
Within populations	165	0.32182	22.92	*P* < 0.001

First model with all populations pooled, second model with populations from different biomes (Caatinga and Brazilian Atlantic Forest) separated, third model with populations from Chapada Diamantina and Planalto da Borborema ecoregions separated, and fourth model with populations from Chapada Diamantina separated from the remaining localities.

Compared with plastid markers, nuclear markers exhibited lower levels of genetic differentiation as estimated by Φ_ST_ (0.114), *G*_ST_ (0.122) and *G*’_ST_ (0.255). An AMOVA also generated different results than for plastid markers, with most of the genetic variation partitioned within (95.42%, *P* < 0.0001) rather than among (4.58%, *P* = 0.087) populations (Table 4). Differentiation between BAF and Caatinga populations and between Chapada Diamantina and Planalto da Borborema was not significant (Table 4). No sign of phylogeographic structure was detected among populations (*P* = 0.329). A Mantel test did not support isolation by distance among populations based on Φ_ST_, *G*_ST_ or *G*′_ST_ (all *P* > 0.05).

**Figure 2:**
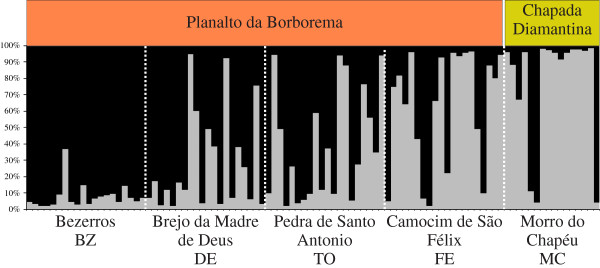
**Genetic assignment results.** Summary of population structure in *Epidendrum secundum* using Bayesian assignment analysis for a *K* = 2 population model. Most individuals from population Bezerros show assignment probabilities associated to cluster 1 (black), whereas specimens from population Morro do Chapéu are mainly classified on cluster 2 (grey). Admixed proportions were found on populations Brejo da Madre de Deus, Pedra de Santo Antonio and Camocim de São Félix. The ecoregion of origin (Chapada Diamantina or Planalto da Borborema) is indicated. See Table 1 for population details.

As shown in Additional file 4: Figure S1, simulations performed in STRUCTURE consistently identified *K* = 2 clusters. Most analyzed individuals showed admixed ancestry, however, and genetic subdivisions among populations were unclear (Figures 1B and 2). Admixture proportions (*Q*) for population BZ showed strong assignment to cluster 2 (average assignment proportion = 0.92; Figure 2), whereas specimens from the southernmost population MC showed assignment proportions (0.77; Figure 2) associated with cluster 1. Intermediate assignment proportions were observed for most individuals from remaining populations (Figure 2).

### Population size reduction and gene flow among *E. secundum* populations

Across all sites and loci, *M*-ratio values ranged from 0.785 to 0.895 (Table 2). The lowest observed critical value (*M*_C_) obtained from simulations performed with different values of parameter θ, proportion of single-step mutations (*pg*) and average size of non-one-step mutations (Δ*g*) was 0.589 (Table 5). According to this *M*c threshold, none of the populations showed signatures of past genetic bottlenecks (Table 2).

**Table 5:** Demographic reduction (bottleneck) results

**Parameter simulations**	**θ**	***pg***	**Δ** ***g***	***M*** **c**
1	0.5	0.1	2.0	0.883
2	0.5	0.3	2.0	0.800
3	0.5	0.1	3.5	0.777
4	0.5	0.3	3.5	0.623
5	2.0	0.1	2.0	0.875
6	2.0	0.3	2.0	0.825
7	2.0	0.1	3.5	0.722
8	2.0	0.3	3.5	0.589
9	10.0	0.1	2.0	0.844
10	10.0	0.3	2.0	0.811
11	10.0	0.1	3.5	0.702
12	10.0	0.3	3.5	0.619

Parameters for the two-phased mutation model (TPM) used to generate critical values of *M*-ratio (*M*c). Theta (θ), proportion of single-step mutations (*pg*) and average size of non one-step mutations (Δ*g*) were used to infer *M*c thresholds.

Using values of genetic differentiation among populations BZ, DE, TO, FE and MC obtained for nuclear (*G*_ST_ = 0.122) and plastid (*G*_ST_ = 0.782) markers, the ratio of pollen flow to seed flow was estimated as 25.85. The value of this ratio suggests that gene flow via pollen in *E. secundum* is > 20 times higher than that occurring via seeds.

### Potential ancient distributions of *E. cinnabarinum* and *E. secundum*

**Figure 3:**
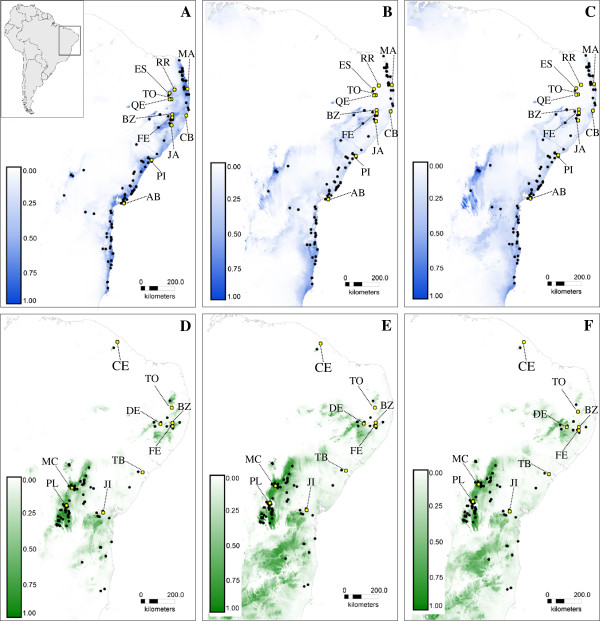
**Environmental envelope models results.** Maps showing environmental envelope models (EEMs) for *Epidendrum cinnabarinum* (blue, AUC = 0.968) and *E. secundum* (green, AUC = 0.969) based on current **(A and D)** and two past scenarios, the Last Glacial Maximum **(B and E)** and Last inter-glacial **(C and F)** using the Maximum Entropy algorithm v. 3.3.3e. Black dots indicate localities used for EEMs analysis, and yellow dots indicates populations sampled for genetic analysis. Darker colors show areas with more suitable predicted conditions, as indicated by blue (*E. cinnabarinum*) and green scales (*E. secundum*). See Table 1 for population details.

Distributions under LIB, LGM and current climatic conditions were successfully estimated for *E. cinnabarinum* and *E. secundum*. As indicated by high area-under-the-curve (AUC) values (0.969 for *E. secundum* and 0.968 for *E. cinnabarinum*), the EEM analysis performed well. Climate variables contributed to both species models, with the *E. cinnabarinum* model additionally adjusted using a geological eras variable. Past distribution ranges differed between the two species, mainly at their peripheral limits. From the LIG to the present, *E. cinnabarinum* exhibited a pronounced decrease in its presence at Chapada Diamantina and other inland mountain ranges, with a more recent expansion towards northern Planalto da Borborema and coastal regions after the LGM (Figure 3A, 3B and 3C). Populations distributed in the southern portion of the northeastern Brazilian seashore did not show marked demographic oscillations. A decrease in the inland distribution of *E. secundum* was also observed, but without any further expansion (Figure 3D, 3E and 3F) as observed for *E. cinnabarinum*. During the LIG and LGM, *E. secundum* was broadly distributed in inland mountains, being present mainly at Cadeia do Espinhaço (comprising Chapada Diamantina and southern mountain ranges) and in western portions of Planalto da Borborema. After the LGM, the species remained broadly distributed only within Chapada Diamantina and mountains to the east within the BAF biome. The model representing current climatic conditions indicated that *E. secundum* has a fragmented distribution within Planalto da Borborema, where most populations are found at the border between Caatinga and BAF biomes. Since the LIG, the distribution of this species has not been continuous between Chapada Diamantina and Planalto da Borborema.

## Discussion

Genetic studies of inselberg species can provide insights into the combined effects of genetic drift and restricted gene flow on evolution and diversification of lineages restricted to disjunct populations [1,2,5]. Phylogeographic studies of inselberg species have indeed confirmed many hypotheses associated with such isolated habitats, such as low levels of gene flow and strong genetic drift [7-11]. In some extreme cases of isolation, within-inselberg interspecific gene flow between congeneric species has been found to be higher than intraspecific gene flow between different inselbergs [7,14]. To understand the effects of long-term disjoint distribution on genetic architecture and evolution of inselberg populations, we carried out a phylogeographic study of two rock-outcrop orchid species showing very similar life traits. The use of plastid markers revealed marked genetic differentiation among populations. In contrast, nuclear genetic differentiation was much lower in *E. secundum*, highlighting the important role of pollen dispersal, and consequently pollination services, in species cohesion. This result has also been observed in other plants [7,40]. Results from EEM analysis of *E. secundum* suggest long-term stability of populations from Chapada Diamantina and the southern portion of Planalto da Borborema (Figure 3D, 3E and 3F); in contrast, results for *E. cinnabarinum* imply a decrease in distribution within Chapada Diamantina, in agreement with its current rarity in this ecoregion. After the LGM, *E. cinnabarinum* expanded its distribution to northern sand dune vegetation communities. This expansion most likely occurred from inland inselberg populations, not from southern coastal ones, as extensive haplotype sharing was found among seashore and inland populations. The joint use of phylogeography and EEMs helped to clarify the origin of genetic diversity and population differentiation in these inselberg species, emphasizing the importance of such studies in terrestrial island-like environments.

### Marked genetic differentiation among inselberg populations

Genetic differentiation among populations of both *E. cinnabarinum* and *E. secundum* was not significantly associated with BAF and Caatinga biomes (Tables 3 and 4). In contrast, extensive haplotype sharing was found among populations distributed in both biomes (Figure 1A and 1B). Haplotype sharing was also found between populations from inselbergs and sand dune vegetation, in agreement with floristic similarities reported in previous studies [41]. Because of their xeric nature, inselbergs can provide suitable conditions for drought tolerant species even within humid forest patches [2]. Inselbergs embedded in mesic forests often harbor xeric plant communities and can be considered refugia for species adapted to drier climatic conditions, highlighting their importance for comparative studies across different biomes [2,4]. Paleontological evidence [42] indicates that alternating dry and wet periods have influenced the BAF distribution of plant species, particularly along the northeastern coast. Inselberg populations within the BAF (populations JA, CB, TB and JI) may thus represent relicts of xeric plant communities that probably extended further towards the coast during drier periods, thereby allowing gene exchange with populations currently placed in the Caatinga biome.

The most outstanding pattern recovered by the use of plastid markers was the deep among-population differentiation within *E. cinnabarinum* and within *E. secundum* (Figure 1A and 1B, Additional files 2 and 3: Table S2 and S3). Even populations separated by a few kilometers exhibited strong differentiation based on the plastidial genome (populations TO and QE, 12 km apart, *F*_ST_ = 0.985; BZ and FE, 18 km apart, *F*_ST_ = 1.000; ES and RR, 34 km apart, *F*_ST_ = 0.727). The significant pairwise genetic differentiation among most populations, the absence of a pattern of isolation by distance, and the lack of significantly phylogeographically structured haplotypes suggest that genetic drift, as observed in other rock outcrop species [7-9,43], has been an important force shaping plastidial genetic variation in *E. cinnabarinum* and *E. secundum*. In finite populations, such as those inhabiting inselbergs and oceanic islands, genetic drift may be the predominant force governing allele frequencies of neutral loci [6,44]. Because effective population size of maternally inherited organelles is expected to be an order of magnitude lower than that of nuclear genes, genetic drift is expected to play a stronger role in shaping frequencies of organellar alleles than those of nuclear alleles [45]. In addition to the spatial dimension, genetic drift includes an intrapopulation temporal component in which a series of subpopulations diverge from one another over time. Fine-scale genetic analysis [43,46] coupled with long-term studies (e.g. [8]) may help to clarify the impact of drift on the genetic structure of inselberg populations.

The genetic structure observed among *E. secundum* populations was higher for plastid than for nuclear markers (Figure 2, Table 4). Most haplotype diversity (63.64%) was found to reside among populations; this result is in contrast to nuclear markers, in which diversity among populations was not significant (4.58%). This difference in genetic structure between genomic compartments was translated into a pollen/seed flow ratio of 25.85, indicating that pollen-mediated gene dispersal is 25-fold more efficient than dispersal through seeds. The inference of extensive pollen flow between *E. secundum* populations is also supported by observations of low inbreeding coefficients (Table 2) and a high proportion of genetic diversity within populations (Table 4). In addition, Bayesian assignment based on nuclear markers indicated intermediate admixture proportions for many individuals and specimens assigned to different nuclear genetic clusters in the same population (Figure 1B); these results also suggest higher levels of gene flow by pollen. Efficient gene flow by pollen is normally expected for food-deceptive orchids [40,47], as pollinators avoid plants in the same patch, thereby promoting pollen flow over long distances and reducing the chances of geitonogamous pollination. In general, low levels of nuclear genetic structure are observed in orchids (reviewed by [48]). Pollen flow has proven to be crucial to the maintenance of species cohesion in other fragmented inselberg species, highlighting the importance of pollinators in promoting gene exchange among rock outcrops [7,8,43].

Despite the strong genetic differentiation observed between populations from Chapada Diamantina and the remaining localities (Table 4), phylogeographic structure was not significant in *E. secundum*. This surprising situation is probably due to ancestral haplotype sharing (Hs5) among most populations, indicating ancient dispersal events. Another explanation for the non-significance of phylogeographic structure is the fact that multiple, phylogenetically unrelated haplotypes are found in the same populations, especially in the Chapada Diamantina ecoregion. Chapada Diamantina is an old and very stable region; demographic changes in peripheral populations, such as those inhabiting the BAF and from mountains south of Chapada Diamantina, may have contributed to the exchange of unrelated haplotypes—a pattern observed in other studies [32,34,49]. Indeed, species distribution modeling shows a broader past geographic distribution for *E. secundum*, with a former range that includes mountains to the south and north of Chapada Diamantina.

### Demographic changes in *E. secundum* and *E. cinnabarinum*

For both species, results obtained using EEMs have shed light on whether haplotype sharing among populations is due to regular gene flow, past long-distance dispersal events or past continuous distribution. For *E. cinnabarinum*, a general pattern of expansion from the inland towards the seashore since the LIG is suggested (Figure 3A, 3B and 3C). This species appears to have been more commonly distributed in Chapada Diamantina during the LIG, showing a further decrease in distribution until the present. *Epidendrum cinnabarinum* is in fact currently very rare in Chapada Diamantina, where its populations are composed of only a few individuals. Colonization of the northern portion of the seashore occurred during the last 20,000 years, probably from Planalto da Borborema populations. The haplotype sharing detected among populations and the low diversity of Mamanguape and Cabo de Santo Agostinho populations are consistent with this hypothesis. Because only ancestral haplotypes are shared among populations, recurrent gene flow is unlikely; in this case, long-distance dispersal may have been responsible for the northern seashore colonization. Results from Behling [42] suggest that xeric vegetation replaced mesic plant communities along the northern portion of the northeastern Brazilian seashore in the last 10,000 years, a trend likely favoring the expansion of *E. cinnabarinum* from inland inselbergs towards dry coastal vegetation zones. Haplotype sharing between inland and coastal populations and the inferred expansion of inland populations towards the seashore have also been observed in other phylogeographic studies along the Brazilian coast, confirming the connection between the BAF and other adjacent biomes such as the Caatinga [32,49], Cerrado [50,51] and Pampas [40,52].

Analysis of EEMs recovered different demographic patterns for *E. secundum* (Figure 3D, 3E, 3F). The long-term persistence of Chapada Diamantina populations is in agreement with the high levels of genetic diversity observed in Morro do Chapéu and Palmeiras populations (Tables 1 and 2) and the absence of bottlenecks (Table 5). The genetic differentiation of Chapada Diamantina and Planalto da Borborema populations was also confirmed by the EEM analysis, which demonstrated a clear discontinuity in *E. secundum* distribution along these two ecoregions in the last 120,000 years. High levels of plant endemism and biodiversity have been recorded for Chapada Diamantina [53,54], and floristic results indicate different species compositions between rock outcrops from Chapada Diamantina and Planalto da Borborema [3]. In addition, phylogeographic studies have indicated the occurrence of older lineages [32] and genetic differentiation among populations [27,34] occurring within Chapada Diamantina. The patterns of genetic diversity found within Chapada Diamantina populations are in agreement with refuge theory, which predicts higher levels of genetic diversity and older lineages to be present in historically climate-stable regions [5,22].

The relictual nature of inselberg populations is also evident in the structure of the haplotype networks (Figure 1C and 1D). Several mutational steps were observed in the networks calculated for *E. cinnabarinum* and *E. secundum*, suggesting substantial periods of isolation among populations of both species. Deep divisions among haplotypes may be reflective of populations with long divergence histories [16], as demonstrated by phylogeographic studies of Caatingan vertebrate animals having fragmented distributions [27,29,31,32]. Divergence times estimated for Caatingan organisms indicate major crown clade splits to have occurred in the Miocene (between 11,000 ka and 5,000 ka), followed by divergence within these major groups during the Pliocene–Pleistocene [20,27,33,34]. These results are also in agreement with the long-term persistence of populations inhabiting inselbergs [1]. Accordingly, lower levels of genetic differentiation have been observed in other *Epidendrum* species inhabiting well-connected landscapes (e.g. sand dunes and swamp vegetation) [40,55].

## Conclusions

The population genetic structure recovered for both *E. cinnabarinum* and *E. secundum* reflects the isolated nature of their inselberg habitats. Similar to the results of studies of other inselberg species [7,9,11,14,15], significant differentiation at plastid loci was observed between populations separated by only a few kilometers (Additional files 2 and 3: Table S2 and S3). Genetic differentiation among populations was not correlated with geographic distance or ecoregions, suggesting that genetic drift may be a significant factor influencing the diversification of inselberg species. According to Porembski et al. [17], inselberg species composition is difficult to predict even over short distances, probably because of stochastic colonization events. Long-term persistence of populations was also supported in our study, suggesting that both species have been restricted to the specific inselberg habitat over long time frames, and have not been reduced to rock outcrops because of climatic instability. The results of EEM analysis indicate that fragmentation and reduction of *E. secundum* distribution has occurred in mountain ranges south of Chapada Diamantina. Future studies should consequently include a broad sampling of this species over adjacent mountain ranges to explore how such distribution oscillations have affected current levels of genetic diversity in these populations. In northeastern Brazil, inselbergs have different mineral origins and are distributed across different biomes and climatic clines. Thus, selection for divergent habitats may also be of great importance during lineage diversification within species under restricted gene flow. Given the contrasting patterns of genetic structure observed between nuclear and plastid markers, cytonuclear incompatibilities may contribute to the first stages of reproductive isolation among divergent lineages. Future efforts should take advantage of next-generation sequencing approaches [56] coupled with experimental studies [57,58] to clarify the role of drift, selection and cytonuclear interactions in the diversification of inselberg species.

## Methods

### Study species

*Epidendrum* L. is the largest genus of Orchidaceae in the Neotropics, with about 1,500 species distributed from the southeastern United States to northern Argentina [59]. The genus contains many species with wide distributions and high morphological diversity [38]. *Epidendrum cinnabarinum* and *E. secundum* are perennial, terrestrial species commonly found on inselbergs whose locations include sea-level to mountain elevations. Both species belong to subgenus *Amphyglottium*[60,61], a group of food-deceptive orchids whose flowers are visited by several butterfly species despite the lack of nectar reward (reviewed by [38]). Although the species are self-compatible, pollinators are necessary for seed set (Pinheiro unpub. res., [62]).

### Sampling design

Samples of *E. cinnabarinum* and *E. secundum* were obtained from 11 and 9 locations, respectively (Table 6, Figure 1A and B). Sample sites covered most of the geographic distribution of these species in the Caatinga, with additional samples collected in the BAF, an adjacent biome with higher levels of humidity and species diversity (perennial mesic forest). To estimate the degree of isolation of inselberg populations, samples were collected from sites ranging from 12 to 1,000 km apart. Most populations were sampled on rock outcrops; *E. cinnabarinum* was also collected in sand dune vegetation (populations MA, PI and AB, Table 6). Both species occurred in sympatry in populations TO, BZ and FE. For molecular analysis, leaf samples were sliced into small pieces and transferred to silica gel for drying. Total genomic DNA was extracted as described by Pinheiro et al. [63].

**Table 6:** Geographic location and habitat description of sampled populations

**Population**	**ID**	**Latitude S**	**Longitude W**	**Altitude (m)**	**Habitat / Biome**^**1**^	**Sample Size**	
						**Nuclear**	**Plastid**
*Epidendrum cinnabarinum*							
Mamanguape	MA	6.789	34.942	50	Sand dune scrub vegetation/BAF	-	24
Serraria	RR	6.827	35.638	553	Rock outcrop within mesic forest enclave/CAA	-	20
Esperança	ES	7.009	35.899	681	Rock outcrop surrounded by SDTF/CAA	-	19
Queimadas	QE	7.351	35.900	497	Rock outcrop surrounded by SDTF/ CAA	-	12
Jaqueira	JA	8.740	35.792	720	Rock outcrop surrounded by forest/BAF	-	14
Cabo de Santo Agostinho	CB	8.217	35.003	116	Rock outcrop surrounded by forest/BAF	-	7
Pirambu	PI	10.61	36.867	97	Shrubby Tabuleiro vegetation/BAF	-	14
Abaeté	AB	12.946	38.357	38	Sand dune scrub vegetation/BAF	-	11
*Epidendrum secundum*							
Maranguape	CE	3.894	38.722	984	Rock outcrop within mesic forest enclave/CAA	-	20
Brejo da Madre de Deus	DE	8.200	36.403	1094	Rock outcrop within mesic forest enclave/CAA	20	20
Serra de Itabaiana	TB	10.739	37.364	572	Rock outcrop surrounded by forest/BAF	-	20
Morro do Chapéu	MC	11.551	41.155	913	Rock outcrop surrounded by SDTF/CAA	16	16
Palmeiras	PL	12.476	41.452	1082	Rock outcrop surrounded by SDTF/CAA	-	20
Serra da Jibóia	JI	12.854	39.476	824	Rock outcrop surrounded by forest/BAF	-	20
Sympatric populations						-	
Pedra de Santo Antonio (C)	TO	7.344	35.798	709	Rock outcrop surrounded by SDTF/CAA	-	15
Pedra de Santo Antonio (S)						20	20
Bezerros (C)	BZ	8.151	35.756	837	Rock outcrop within mesic forest enclave/CAA	-	21
Bezerros (S)						20	20
Camocim de São Félix (C)	FE	8.328	35.754	718	Rock outcrop surrounded by SDTF/CAA	-	20
Camocim de São Félix (S)						20	18
Total						96	351

^1^SDTF – Seasonal Dry Tropical Forest;

Populations sampled with their identification code (ID), elevation above sea level, habitat description and sample size, analysed for nuclear and plastid markers in *Epidendrum cinnabarinum* (C) and *E. secundum* (S) from Northeast Brazil, distributed in the Caatinga (CAA) and Brazilian Atlantic Forest (BAF) biomes. Populations are indicated as shown on the maps in Figure 1A and B.

### Molecular markers and genotyping assays

Six plastid microsatellite loci were used to genotype samples from both species (Epcp02, Epcp04, Epcp05, Epcp07, Epcp08 and Epcp09 [64]). For *E. cinnabarinum*, the intergenic *rps16–trnK* region was also sequenced to detect a 16-bp insertion. Samples of *E. secundum* from five populations (Table 6) were also analyzed at nuclear microsatellite loci Eff06, Eff26, Eff45 [63], Eff48 (forward primer 5′-TGACCGTTTGAACCTTTTGGT-3′; reverse primer 5′-ATCCAGGCATGAGCAGCA-3′), Epp96 [65] and Lspe-3 [66]. Nuclear microsatellites were not amplified for *E. cinnabarinum* samples owing to the polyploid origin of this species (2*n* = 240). All polymerase chain reaction (PCR) amplifications were performed in an Applied Biosystems 2700 thermocycler (Applied Biosystems, Foster City, CA, USA) following the protocol described by Pinheiro et al. [63]. Microsatellite alleles were resolved on an ABI 3130 Genetic Analyzer automated sequencer and were sized with LIZ (500) standard using GENEMAPPER v. 4.1 software (Applied Biosystems).

### Genetic diversity of sampled populations

All sampled populations were characterized for levels of diversity based on plastid DNA markers. The number of haplotypes in each population, haplotype diversity and haplotype richness were estimated using RAREFAC v. 3.5 software [[67]]. Estimates of haplotype richness were corrected for differences in sample size using the rarefaction method.

Nuclear microsatellite diversity of five *E. secundum* populations was characterized according to number of alleles, number of private alleles, allelic richness, private allelic richness, expected heterozygosity and inbreeding coefficient [68], which were calculated using the programs MSA v. 4.05 [69] and HP-RARE v. 1.0 [70]. Departures from Hardy–Weinberg Equilibrium of within-population inbreeding coefficients were identified using exact tests in GENEPOP v. 4.0 [71]. The microsatellite data set was tested for genotyping errors due to stuttering, short allele dominance and null alleles by means of a Monte Carlo simulation of expected allele-size differences as implemented in MICRO-CHECKER [39].

### Plastid genetic structuring

The geographical structure of genetic variation in plastid DNA was investigated for both species through several approaches. A median-joining network [72] based on plastid DNA was constructed using the program NETWORK v. 4.5.1.0 (http://www.fluxus-engineering.com). To assess whether observed genetic differentiation was due to drift, we tested the hypothesis that *G*_ST_ = *R*_ST_[73] (where Slatkin’s *R*_ST_[74] estimates the contribution of stepwise-like mutations to genetic differentiation) following Pons and Petit [75] using the program PERMUT/CpSSR. To test for the presence of phylogeographic structure among populations (i.e. *R*_ST_ significantly larger than *G*_ST_), 10,000 permutations of *R*_ST_ values were performed. Partitioning of genetic diversity within and among populations and between populations from BAF (coastal) and Caatinga (inland) regions was assessed by AMOVA. For *E. secundum*, two additional models were considered, one corresponding to differentiation between populations from Chapada Diamantina and Planalto da Borborema, and the other considering populations from Chapada Diamantina separated from remaining localities. Genetic differentiation between populations was also measured by pairwise comparisons of *F*_ST_ using the program ARLEQUIN v. 3.5. Finally, the hypothesis that populations were differentiated because of isolation by distance was tested by assessing the correlation between pairwise geographic distances and pairwise *F*_ST_ values using a Mantel test in the program GENEPOP v. 4.0. Correlation significance was estimated after performing 10,000 permutations between pairwise geographic distance and pairwise genetic differentiation matrices.

### Nuclear genetic structuring

Nuclear genetic differentiation among populations of *E. secundum* was measured using the unbiased estimator of relative differentiation *G*_ST_[76] and the standardized genetic differentiation measure *G*′_ST_[77]. Partitioning of genetic diversity was examined at different hierarchical levels using AMOVA as implemented in ARLEQUIN v. 3.5. The hypothesis that populations were differentiated because of isolation by distance was tested by assessing the correlation between pairwise geographic distance and pairwise *G*′_ST_ values using a Mantel test in GENEPOP v. 4.0, similar to the approach used for plastid markers. In addition, to test for the presence of phylogeographic structure among populations, *R*_ST_ was calculated by permuting allele sizes among alleles for 10,000 permutations using the program SPAGEDI v. 1.3d [78], and was then compared with *F*_ST_ values. Phylogeographic structure was inferred when *R*_ST_ was significantly larger than *F*_ST_.

Bayesian assignment analysis (in STRUCTURE v. 2.3.3 [79]) was used to assign individuals to genetic clusters (*K*) and to estimate admixture proportions (*Q*) for each individual. A set of models was chosen in which individuals had admixed ancestries and correlated allele frequencies. Ten replicate runs were completed for each *K* value for *K* = 1–10. Markov chain Monte Carlo runs consisted of 600,000 generations after an initial burn-in of 250,000 generations. The most probable number of genetic clusters (*K*) present in the data were defined following Evanno et al. [80] using the program Structure Harvester v. 6.0 [81].

### Demographic analyses

Recent population size reductions (i.e. genetic bottlenecks) were tested in *E. secundum* with nuclear microsatellite-based *M*-ratios [82] using ARLEQUIN v. 3.5. Significance for each population was assessed by comparisons of *M*-ratios and critical values (*M*_C_ values) obtained by simulating the distribution of *M*-ratios under specific demographic and mutational conditions using the software CRITICAL_M.EXE (http://swfsc.noaa.gov/textblock.aspx?Division=FED&id=3298). The critical value *M*_C_ is set at the lower 5% tail of this distribution, and bottlenecks are detected when the *M*-ratio value is below the calculated *M*_C_ threshold. Different values of *M*_C_ were simulated by modifying θ (0.5, 2.0 and 10.0), *pg* (0.1 and 0.3) and Δ*g* (2.0 and 3.5). Low *M*_C_ values are more conservative as a bottleneck must be of greater intensity to drop below this level [82]; for this reason, the lowest obtained *M*_C_ value was used to check bottleneck significance.

To examine the relative contribution of pollen vs. seed flow to total gene flow among *E. secundum* populations, the parameter *G*_ST_ for nuclear and plastid loci was compared based on populations genotyped for both plastid and nuclear markers (TO, DE, BZ, FE and MC). Pollen/seed flow ratio was estimated following Ennos [83] and Petit et al. [84] using Equation 1 presented by the latter authors.

### Environmental envelope models for *E. cinnabarinum* and *E. secundum*

To test the hypothesis that inselbergs were refugia through late Quaternary climate cycles, we projected EEMs from the current situation onto late Quaternary scenarios. We used the maximum entropy algorithm of Maxent v. 3.3.3e [85,86] to obtain current and past distributions. We used current and past bioclimatic variables derived from monthly temperature and rainfall (Bioclim scheme). Current data were obtained from WorldClim [87]. Two past scenarios were considered: LIG (~120,000–140,000 years BP), based on Otto-Bliesner et al. [88], and LGM (~21,000 years BP), based on Paleoclimate Modelling Intercomparison Project Phase II [89] considering the CCSM3 model [90]. To incorporate geological differentiation between outcrops, we also used three variables derived from Schenk et al. [91]. Geological variables were major geological types (sedimentary/igneous and metamorphic rocks), eras and geological age. Major geological types and eras were categorical and age was continuous. All variables were adjusted to a spatial resolution of 30 arcsec. Highly correlated variables were removed, and jackknifing was used to estimate variable importance. The final models were obtained considering only variables with contributions to AUC higher than 75%. Five model replicates were run for each one of the presence-only methods, with 75% of occurrences used for calibration and different subsets (25%) used for validation. Mean AUC was used to assess the performance of the models [92], where 1 was the maximum prediction and 0.5 suggested a random prediction. Past distributions were estimated by projecting the current relationship onto scenarios of past climate, assuming that current relationships between climate and distribution were maintained.

We obtained 226 *E. cinnabarinum* and 242 *E. secundum* occurrences from our field GPS records and georeferenced herbarium data extracted from the *species*Link project (http://splink.cria.org.br). All points were verified with Google Earth to ensure localities were not placed in heavily urbanized areas. The localities used for EEM analysis are available from the authors upon request to avoid illegal plant collecting.

## Abbreviations

EEMs: Environmental envelope models; LGM: Last Glacial Maximum; BAF: Brazilian Atlantic Forest; LIG: Last Interglacial; AUC: Area under the curve.

## Competing interests

The authors declare that they have no competing interests.

## Authors’ contributions

FP participated in the study design, carried out the molecular genetic studies and drafted the manuscript. SC and CPS participated in the study design, and FB coordinated the study. LPF participated in the field work and sampling strategy design. MFF contributed to chemical supply procurement and analysis. DDM conducted the EEM analysis. All authors help to draft the manuscript, and read and approved the final version.

## Supplementary Material

Additional file 1: Table S1 Description of 10 chloroplast microsatellite haplotypes of *Epidendrum cinnabarinum* and 12 haplotypes of *E. secundum*, characterized at six cpSSR loci. For *E. cinnabarinum*, the presence (1) and absence (0) of a 16 bp fragment from intergenic region *rps16–trnK* is also reported. The frequency of occurrence (*n*) in total collection screened is indicated for both species. Click here for file

Additional file 2: Table S2 Pairwise comparisons of *F*_ST_ between populations of *Epidendrum cinnabarinum* based on plastid markers. Click here for file

Additional file 3: Table S3 Pairwise comparisons of *F*_ST_ between populations of *Epidendrum secundum* based on plastid markers. Click here for file

Additional file 4: Figure S1 Magnitude of Δ*K* from STRUCTURE analysis as a function of *K* (number of genetic groups, details in Methods) calculated according to the simulations described by Evanno et al. [80]. The modal value of these distributions indicates the true *K* or the uppermost level of structure—in the present case, two genetic clusters. Click here for file
