# Supplementary material for: Rock outcrop orchids reveal the genetic connectivity and diversity of inselbergs of northeastern Brazil
Source: BMC Evol Biol. 2014 Mar 15;14:49. doi: 10.1186/1471-2148-14-49 (PMC4004418; doi:10.1186/1471-2148-14-49)
Supplement: Additional file 1: Table S1 — Description of 10 chloroplast microsatellite haplotypes of Epidendrum cinnabarinum and 12 haplotypes of E. secundum, characterized at six cpSSR loci. For E. cinnabarinum, the presence (1) and absence (0) of a 16 bp fragment from intergenic region rps16–trnK is also reported. The frequency of occurrence (n) in total collection screened is indicated for both species. [file 1471-2148-14-49-S1.doc]

# **Table S1.** Description of 10 chloroplast microsatellite haplotypes of *Epidendrum cinnabarinum* and 12 haplotypes of *E. secundum*, characterized at six cpSSR loci. For *E. cinnabarinum*, the presence (1) and absence (0) of a 16 bp fragment from intergenic region *rps16–trnK* is also reported. The frequency of occurrence (*n*) in total collection screened is indicated for both species.

| Haplotypes | Epcp02 | Epcp04 | Epcp05 | Epcp07 | Epcp08 | Epcp09 | *rps16–trnK* | *n* |
| --- | --- | --- | --- | --- | --- | --- | --- | --- |
| *E. cinnabarinum* |  |  |  |  |  |  |  |  |
| Hc1 | 267 | 113 | 139 | 216 | 87 | 242 | 1 | 83 |
| Hc2 | 267 | 113 | 139 | 216 | 87 | 241 | 1 | 8 |
| Hc3 | 267 | 113 | 140 | 216 | 90 | 241 | 0 | 3 |
| Hc4 | 267 | 113 | 140 | 216 | 90 | 242 | 0 | 3 |
| Hc5 | 268 | 113 | 140 | 216 | 90 | 241 | 0 | 48 |
| Hc6 | 268 | 113 | 140 | 216 | 90 | 242 | 0 | 6 |
| Hc7 | 269 | 113 | 140 | 216 | 90 | 241 | 0 | 11 |
| Hc8 | 270 | 113 | 139 | 216 | 93 | 242 | 0 | 6 |
| Hc9 | 270 | 113 | 140 | 216 | 90 | 242 | 0 | 2 |
| Hc10 | 271 | 113 | 139 | 216 | 93 | 242 | 0 | 7 |
| Total |  |  |  |  |  |  |  | 177 |
| *E. secundum* |  |  |  |  |  |  |  |  |
| Hs1 | 270 | 112 | 143 | 218 | 90 | 249 | - | 9 |
| Hs2 | 270 | 113 | 143 | 216 | 93 | 243 | - | 4 |
| Hs3 | 271 | 111 | 143 | 218 | 89 | 249 | - | 2 |
| Hs4 | 271 | 112 | 143 | 216 | 93 | 243 | - | 3 |
| Hs5 | 271 | 113 | 143 | 216 | 93 | 243 | - | 95 |
| Hs6 | 271 | 113 | 143 | 216 | 93 | 242 | - | 3 |
| Hs7 | 271 | 113 | 143 | 216 | 94 | 243 | - | 3 |
| Hs8 | 272 | 112 | 143 | 216 | 93 | 243 | - | 2 |
| Hs9 | 272 | 112 | 143 | 218 | 89 | 249 | - | 9 |
| Hs10 | 272 | 113 | 143 | 216 | 93 | 242 | - | 4 |
| Hs11 | 272 | 113 | 143 | 216 | 93 | 243 |  | 37 |
| Hs12 | 273 | 112 | 143 | 218 | 89 | 249 | - | 3 |
| Total |  |  |  |  |  |  |  | 174 |
