# Supplementary material for: Rock outcrop orchids reveal the genetic connectivity and diversity of inselbergs of northeastern Brazil
Source: BMC Evol Biol. 2014 Mar 15;14:49. doi: 10.1186/1471-2148-14-49 (PMC4004418; doi:10.1186/1471-2148-14-49)
Supplement: Additional file 2: Table S2 — Pairwise comparisons of FST between populations of Epidendrum cinnabarinum based on plastid markers. [file 1471-2148-14-49-S2.doc]

**Table S2.** Pairwise comparisons of *F*_ST_ between populations of *Epidendrum cinnabarinum* based on plastid markers. See Table 1 for population identification.

|  | AB | MA | PI | CB | JA | BZ | ES | FE | QE | RR | TO |
| --- | --- | --- | --- | --- | --- | --- | --- | --- | --- | --- | --- |
| AB | * |  |  |  |  |  |  |  |  |  |  |
| MA | **1.000** | * |  |  |  |  |  |  |  |  |  |
| PI | **0.650** | **0.893** | * |  |  |  |  |  |  |  |  |
| CB | **1.000** | **1.000** | **0.480** | * |  |  |  |  |  |  |  |
| JA | **0.758** | 0.227 | **0.559** | **0.704** | * |  |  |  |  |  |  |
| BZ | **1.000** | 0.000 | **0.884** | **1.000** | 0.207 | * |  |  |  |  |  |
| ES | **0.459** | **0.812** | 0.209 | 0.162 | **0.508** | **0.800** | * |  |  |  |  |
| FE | **0.455** | **0.794** | 0.228 | 0.193 | **0.491** | **0.781** | 0.010 | * |  |  |  |
| QE | **0.919** | **0.988** | **0.476** | -0.051 | **0.731** | **0.987** | 0.185 | 0.217 | * |  |  |
| RR | **0.929** | **0.395** | **0.812** | **0.918** | 0.130 | **0.375** | **0.727** | **0.710** | **0.917** | * |  |
| TO | **1.000** | 0.000 | **0.862** | **1.000** | 0.162 | 0.000 | **0.770** | **0.750** | **0.985** | 0.329 | * |

Values given in bold are significant at *P* < 0.005.
