# Supplementary material for: Rock outcrop orchids reveal the genetic connectivity and diversity of inselbergs of northeastern Brazil
Source: BMC Evol Biol. 2014 Mar 15;14:49. doi: 10.1186/1471-2148-14-49 (PMC4004418; doi:10.1186/1471-2148-14-49)
Supplement: Additional file 3: Table S3 — Pairwise comparisons of FST between populations of Epidendrum secundum based on plastid markers. [file 1471-2148-14-49-S3.doc]

**Table S3.** Pairwise comparisons of *F*_ST_ between populations of *Epidendrum secundum* based on plastid markers. See Table 1 for population identification.

|  | PL | MC | JI | TB | CE | TO | FE | BZ | DE |
| --- | --- | --- | --- | --- | --- | --- | --- | --- | --- |
| PL | * |  |  |  |  |  |  |  |  |
| MC | **0.158** | * |  |  |  |  |  |  |  |
| JI | **0.416** | **0.498** | * |  |  |  |  |  |  |
| TB | **0.500** | **0.591** | 0.043 | * |  |  |  |  |  |
| CE | **0.382** | **0.461** | 0.054 | 0.089 | * |  |  |  |  |
| TO | **0.555** | **0.647** | 0.105 | 0.000 | 0.157 | * |  |  |  |
| FE | **0.586** | **0.632** | **0.859** | **0.947** | **0.823** | **1.000** | * |  |  |
| BZ | **0.555** | **0.647** | 0.105 | 0.000 | 0.157 | 0.000 | **1.000** | * |  |
| DE | **0.547** | **0.591** | **0.807** | **0.895** | **0.772** | **0.947** | -0.005 | **0.947** | * |

Values given in bold are significant at *P* < 0.005.
