# Supplementary material for: Rock outcrop orchids reveal the genetic connectivity and diversity of inselbergs of northeastern Brazil
Source: BMC Evol Biol. 2014 Mar 15;14:49. doi: 10.1186/1471-2148-14-49 (PMC4004418; doi:10.1186/1471-2148-14-49)
Supplement: Additional file 4: Figure S1 — Magnitude of ΔK from STRUCTURE analysis as a function of K (number of genetic groups, details in Methods) calculated according to the simulations described by Evanno et al. [80]. The modal value of these distributions indicates the true K or the uppermost level of structure—in the present case, two genetic clusters. [file 1471-2148-14-49-S4.pdf]

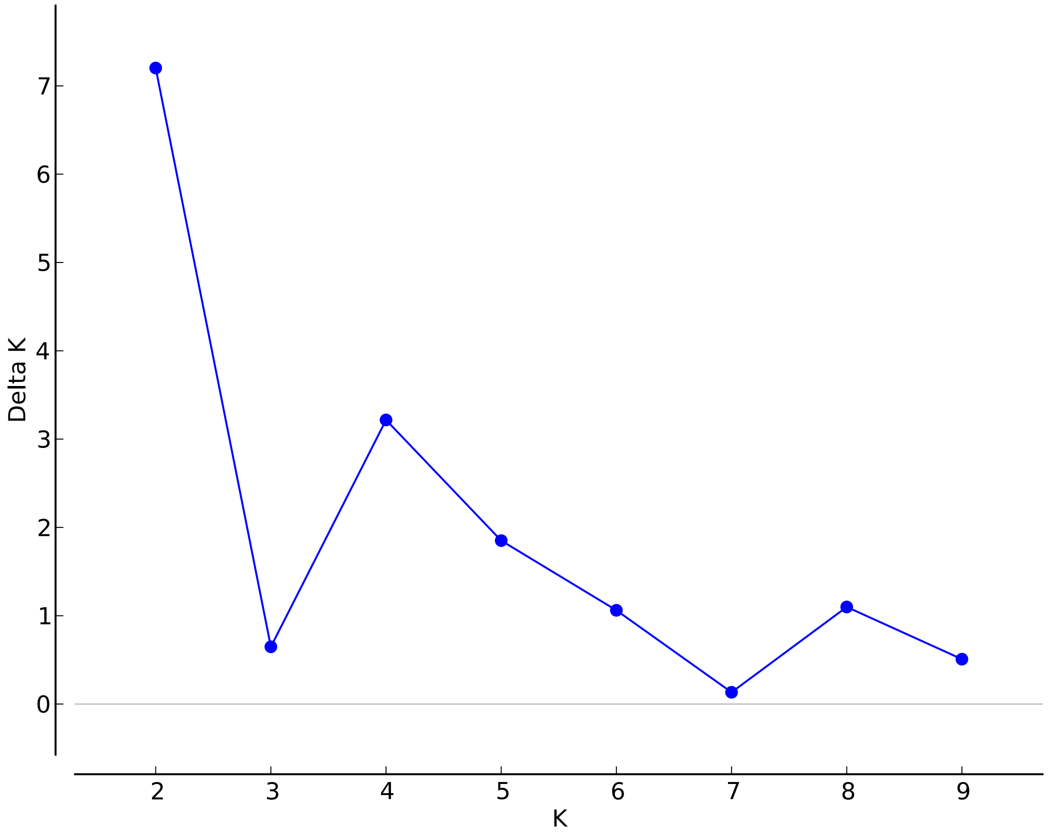

Figure S1. Magnitude of ΔK from STRUCTURE analysis as a function of *K* (number of genetic groups, details in Material and Methods), calculated according to the simulations described by Evanno et al. (2005). The modal value of these distributions indicates the true *K* or the uppermost level of structure, in the present case, two genetic clusters.
